# Interactions of calmodulin kinase II with the dopamine transporter facilitate cocaine-induced enhancement of evoked dopamine release

**DOI:** 10.1038/s41398-023-02493-4

**Published:** 2023-06-13

**Authors:** Jacqueline D. Keighron, Jordi Bonaventura, Yang Li, Jae-Won Yang, Emily M. DeMarco, Melinda Hersey, Jianjing Cao, Walter Sandtner, Michael Michaelides, Harald H. Sitte, Amy Hauck Newman, Gianluigi Tanda

**Affiliations:** 1 Medication Development Program, National Institute on Drug Abuse, Intramural Research Program, Baltimore, MD USA; 2 Biobehavioral Imaging & Molecular Neuropsychopharmacology Unit, Neuroimaging Research Branch, National Institute on Drug Abuse, Intramural Research Program, Baltimore, MD USA; 3 Center for Physiology and Pharmacology, Medical University of Vienna, Vienna, Austria; 4 Molecular Targets and Medications Discovery Branch, National Institute on Drug Abuse, Intramural Research Program, Baltimore, MD USA; 5 Present Address: Department of Biological and Chemical Science, New York Institute of Technology, Old Westbury, NY USA; 6 Present Address: Department of Pathology and Experimental Therapeutics, Institut de Neurociències, Universitat de Barcelona, L’Hospitalet de Llobregat, Catalonia Spain

**Keywords:** Molecular neuroscience, Addiction

## Abstract

Typical and atypical dopamine uptake inhibitors (DUIs) prefer distinct conformations of the dopamine transporter (DAT) to form ligand-transporter complexes, resulting in markedly different effects on behavior, neurochemistry, and potential for addiction. Here we show that cocaine and cocaine-like typical psychostimulants elicit changes in DA dynamics distinct from those elicited by atypical DUIs, as measured via voltammetry procedures. While both classes of DUIs reduced DA clearance rate, an effect significantly related to their DAT affinity, only typical DUIs elicited a significant stimulation of evoked DA release, an effect unrelated to their DAT affinity, which suggests a mechanism of action other than or in addition to DAT blockade. When given in combination, typical DUIs enhance the stimulatory effects of cocaine on evoked DA release while atypical DUIs blunt them. Pretreatments with an inhibitor of CaMKIIα, a kinase that interacts with DAT and that regulates synapsin phosphorylation and mobilization of reserve pools of DA vesicles, blunted the effects of cocaine on evoked DA release. Our results suggest a role for CaMKIIα in modulating the effects of cocaine on evoked DA release without affecting cocaine inhibition of DA reuptake. This effect is related to a specific DAT conformation stabilized by cocaine. Moreover, atypical DUIs, which prefer a distinct DAT conformation, blunt cocaine’s neurochemical and behavioral effects, indicating a unique mechanism underlying their potential as medications for treating psychostimulant use disorder.

## Introduction

Blockade of the dopamine transporter (DAT) [1, 2] and the related boost in dopamine (DA) transmission is central to the behavioral and reinforcing actions of cocaine and other misused psychostimulants [3]. Cocaine has been shown to indirectly increase brain DA levels by inhibiting DAT-mediated clearance of DA from the synapse following quantal release (exocytosis) in rodents and in humans [4, 5]. Importantly, cocaine does not produce an increase in DA via a DAT-mediated, Ca^2+^-independent efflux of DA as seen with a subset of addictive psychostimulants, like amphetamines [6–8]. In spite of early suggestions that all DAT blockers would have the addictive potential [3], atypical DAT blockers with behavioral and neurochemical profiles different from typical addictive psychostimulants have been discovered [9–11]. Typical and atypical DA uptake inhibitors (DUIs), though binding to overlapping DAT sites, have been shown to prefer or stabilize distinct, outward- vs. inward-facing, DAT conformations [12–14]. Preference for different DAT conformations has been suggested to play a role in the pharmacological profile and addictive potential of DAT blockers [12, 15–18], and also in the distribution of DAT in dopaminergic terminals [19]. In this study we assessed the influence of the administration of typical (cocaine-like) or atypical (not cocaine-like) DAT blockers on DA dynamics, phasic, evoked DA release, and DA clearance rate, in the nucleus accumbens shell (NAS) of mice, using fast-scan cyclic voltammetry (FSCV) procedures [20]. Also, using electrophysiology procedures we assessed potential differences between typical and atypical DAT blockers in their kinetics of DAT-binding association/dissociation rates [21], which could be related to DAT conformation. Due to the calcium dependency of cocaine-induced stimulation of DA release, under the same FSCV experimental conditions, changes in free calcium fluctuations were assessed for typical and atypical DAT blockers using fiber photometry procedures. DAT function may be influenced by its interactions with other neuronal membrane proteins, small molecules, and cations [22–25]. Previous reports have attributed cocaine-induced enhancement of evoked DA release to an increased availability of DA-containing large dense-core vesicles (LDCVs) associated with the synapsin-mediated reserve pool of vesicles under resting conditions [26, 27]. Thus, we tested the effects of intracerebroventricular (ICV) administration of KN93 [28], an inhibitor of CaMKIIα, a kinase that functionally interacts with DAT [29, 30] and is involved in the regulation of synapsin activity [31], on the cocaine-induced enhancement of phasic DA release. We hypothesized that distinct conformations of DAT, stabilized by binding with typical or atypical DUIs [15, 16], would potentially facilitate or hinder DAT interactions with CaMKIIα, thus regulating phosphorylation of synapsins, mobilization of the reserve pool of DA vesicles [27], and enhancement of evoked DA release.

## Methods

### Subjects

Adult male Swiss-Webster mice (Charles River, MA), experimentally naïve at the start of the study weighing 30–40 g and ~8–12 weeks old, were housed in groups of four and had free access to food and water. The housing rooms were temperature and humidity controlled and maintained on a 12 h light/dark cycle. Experiments were conducted during the light phase. The housing facilities were fully accredited by AAALAC International, and all experimentation was conducted in accordance with the Guidelines of the Animal Care and Use Committee of the Intramural Research Program, National Institute on Drug Abuse, National Institutes of Health, and the *Guide for Care and Use of Laboratory Animals* [32]. Mice were not used in more than one experiment.

### Drug preparation

(-)Cocaine HCl and methylphenidate were from Sigma-Aldrich, St. Louis, MO; KN93, KN92, and desipramine hydrochloride was from Tocris, Minneapolis, MN; WIN 35428 (β-CFT) was from the National Institute on Drug Abuse, Drug Supply Program; JHW 007 hydrochloride and JJC8-091oxalate were synthesized in the Medicinal Chemistry Section, NIDA IRP, according to previously reported procedures [33, 34]. Drugs were dissolved in saline (0.9% NaCl) [(-)cocaine HCl, WIN 35428, and methylphenidate], sterile water [JHW 007], or a vehicle containing 10% DMSO, 15% Tween 80, and sterile water [JJC8-091] and were injected in a volume of 10 ml/kg i.p. Injections of saline (10 ml/kg i.p.) served as vehicle controls. DMSO was used to dissolve KN92 and KN93 which were injected in a volume of 1 µl, at 0.5 µl/min (i.c.v. to the lateral ventricle), similar injections of DMSO served as vehicle control. In the “Results” section and in the figures, doses of drugs are expressed as micromoles (µmol)/kg, which can be converted to mg/kg using the information provided in Table 1.

**Table 1:** Conversions between drug doses expressed as mg/kg or μmol/kg.

Compound	Affinity for DAT *K*_D_ (nM)	Molecular weight (g/mol)	Dose range (mg/kg)	Dose range (μmol/kg)
Cocaine [55]	76.6	339.81	0.5–56	1.5–165
JHW 007 [56]	12	421.96	0.5–17	1.2–40.3
R-Modafinil [57]	3050	273.35	5–100	18.3–366
WIN 35428 [58]	5.24	427.42	0.032–3.2	0.075–7.5
Methylphenidate [14]	21.2	269.77	0.1–56	0.37–207
Desipramine [59]	5200	302.84	3.2–100	10.6–330
JJC8-016 [20, 33, 57]	116	377	1–56	2.7–149
JJC8-088 [20, 33]	2.53	678.7	0.5–100	0.74–147
JJC8-091 [20, 33]	289	611.61	5–56	8.2–92

$$\frac{{\mu mol}}{{kg}} = \frac{{mg}}{{kg}} \ast \frac{{1\;g}}{{1000\;mg}} \ast \frac{1}{{MW}} \ast \frac{{1e6\;\mu mol}}{{mol}}$$

### Surgery

Mice were anesthetized with 1.2 g/kg urethane (i.p.) (Sigma-Aldrich, St. Louis, MO). Mice were then placed in a stereotaxic apparatus where the skull was exposed, and holes were drilled to expose the dura. All mice were implanted with bipolar tungsten stimulation electrodes in the medial forebrain bundle (posterior −1.5 mm, lateral ± 1.0 mm, and ventral –4.5 mm); stimulation was tested by applying a train of 24 pulses of 180 µA, 60 Hz, 4 ms is duration which produced a detectable movement of the whiskers.

Subjects used for FSCV experiments were also implanted with an Ag/AgCl reference electrode secured by a screw in the contralateral hemisphere, and a carbon fiber microelectrode was slowly lowered to a final position in the NAS (anterior +1.5 mm, lateral ±1.3 mm, and ventral −4.8 to −5.2 mm) while testing the DA response to a stimulus to ensure a robust dopamine signal was found. In a subset of subjects, a 19-gauge cannula (PlasticsOne, Roanoke, VA) was implanted into the ipsilateral lateral ventricle (anterior ±0 mm, lateral ±1.0 mm, ventral −3.0 mm) to allow for i.c.v. delivery of drugs. At the conclusion of the experiment, the working electrode placement was marked by applying 10 V cathodically for 30 s to create a lesion detectable during the histology procedure.

In addition to the stimulation electrode subjects used for fiber photometry experiments were implanted with an optical fiber in the NAS (anterior +1.5 mm, lateral ±1.3 mm, and ventral −4.8 to −5.2 mm).

At the conclusion of the experiment each subject was euthanized, and the brain was retained for dissection using a cryostat to confirm the placement of all implants. From a total of 96 animals, 4 animals, for which the histology did not show proper placement, were excluded from the data presented in this manuscript.

### Fast scan cyclic voltammetry

Phasic, evoked DA release, and DA clearance rate were measured by FSCV procedures, following those of recently published work from our laboratory [20]. Briefly, glass-sealed 100 µm carbon fiber microelectrodes were pre-calibrated with known concentrations of dopamine and changes in pH to allow for a principal component analysis (PCA) of the raw data using HDCV (UNC, Chapel Hill, NC). Dopamine was identified by cyclic voltammogram using a voltage scan from −0.4 to 1.3 V at 400 V/s. During the experiment, an external stimulus was applied using the tungsten electrode every 5 min comprised of 24 pulses 4 ms in width at 60 Hz and 180 µA.

PCA data were then analyzed to determine the DA_Max_ and DA clearance rate using a custom macro written in Igor Carbon Pro which identified peaks greater than 3× root mean square noise and fit to Eq. (1) where DA_Max_ represents the peak DA concentration measured, *k* is the rate constant, and *t* is time [35, 36].

1 $${{{\mathrm{DA}}}}({{{\mathrm{t}}}}) = {{{\mathrm{DA}}}}_{{{{\mathrm{Max}}}}}{{{\mathrm{e}}}}^{ - {{{k}}}({{{t}}} - {{{t}}}_0)}$$

**Fig. 1:**
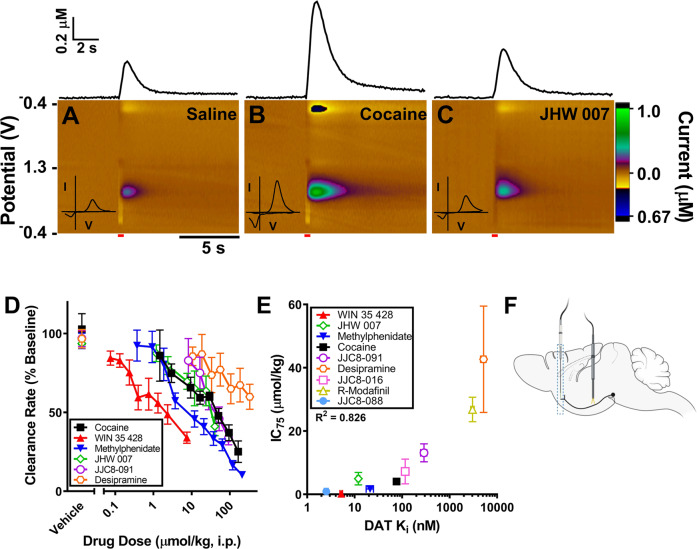
Typical and atypical dopamine uptake inhibitors have different effects on elicited DA. **A**–**C** The stimulus elicited DA peak measured by FSCV is shown as a representative colorplot for **A** saline, **B** 29 μmol/kg cocaine, and **C** 24 μmol/kg JHW 007. In each panel, the inset cyclic voltammogram identifies the substance being measured as dopamine while the trace above each panel represents the change in DA concentration as a function of time. The red bar in each panel represents the time and duration of the electrical stimulation of the MFB. **D** The change in the clearance rate of DA produced by each DUI tested (cocaine, *n* = 4; methylphenidate, *n* = 8; WIN 35428, *n* = 4, JHW007, *n* = 6; JJC8-091, *n* = 5; desipramine, *n* = 5) is shown as a function of drug dose. Saline (*n* = 8), the control vehicle, had no significant effect on DA clearance rate. **E** An inhibition constant for a series of DUIs was generated from the data in (**D**) and from data collected under the same experimental conditions, including *R*-modafinil, JJC8-016 and JJC8-088, in a recent publication [20]. The ability of each DUI to prevent DA clearance was found to significantly correlate to DAT affinity of the DUIs. **F** The experimental setup showing the recording electrode in the NAS and the stimulation electrode in the medial forebrain bundle. Error bars represent mean ± SEM.

In Fig. 1, an IC_75_ was chosen because not all DAT inhibitors can produce a 50% change in DA clearance rate even at doses as high as 100 mg/kg, while higher doses of JHW 007 produce other systemic side effects.

### Fiber photometry

Animals were anesthetized with a mixture of ketamine/xylazine (60/10 mg/kg i.p.) and placed on a stereotaxic apparatus (Kopf, Germany). An adeno-associated virus (AAV5) expressing GCaMP6f under the control of a hSyn promoter [Addgene: pAAV.Syn.GCaMP6f.WPRE.SV40 (#100837)] was injected in the VTA (unilaterally, 0.5 μl) using a Hamilton Neuros 33 G syringe at a flow rate of 50 nl min^−1^. The following coordinates [37], were used to target the VTA: AP = −3.3, ML = ±0.5, DV = −4.7. One to two months after viral delivery, a stimulating electrode and a fiber optic were lowered into the brain as described above. A real-time signal processor (RX8; Tucker-Davis Technologies), running software that was custom-designed to drive a modulated LED input (488 nM), the output signal was filtered through a 535 nm low pass filter and projected onto a photodetector (model 2151 femtowatt photoreceiver; Newport) and signals were collected (sample rate 381 Hz) and digitally demodulated and amplified through a lock-in amplifier. The power output of the system was tested with a power meter at the start of each experimental session. The RX8 interface also collected TTL inputs from the controller of the stimulating electrode to localize the stimulation events applied using the tungsten electrode every 5 min comprised of 24 pulses 4 ms in width at 60 Hz and 180 µA (see above). Digitized signals were processed using custom code written in Matlab and Igor Carbon Pro and changes in fluorescence were calculated as (*F*−*F*_0_)/*F*_0_ where *F* is the fluorescence detected at any given time point and *F*_0_ is the mean fluorescence of a 10 s period before stimulation.

### Electrophysiology: DAT *k*_off_ analysis

For these experiments, ATCC, and human embryonic kidney cells 293 (HEK293) were used; authentication was not performed. Mycoplasma contamination was regularly tested and could be ruled out for all cell lines used in this study. HEK293 cells stably expressing DAT were seeded at low density 24 h before recording. The current induced by substrates were voltage clamped with an Axopatch 200B amplifier and recorded in the whole cell patch clamp configuration. The resistance of the electrode was between 2 and 4 MΩ. The pipette was filled with internal solution (K- gluconate 133 mM, NaCl 6 mM, CaCl_2_ 1 mM, MgCl_2_ 0.7 mM, EGTA 10 mM, HEPES 10 mM pH adjusted to 7.2 using KOH). The external solution contained NaCl 140 mM, KCl 3 mM, CaCl_2_ 2.5 mM, MgCl_2_ 2 mM, glucose 20 mM, HEPES 10 mM pH adjusted to 7.4 with NaOH. The cells were continuously superfused with external solutions or solutions containing different drug concentrations utilizing a DAT-12 device (Adams & List, Westbury, NY, USA). The holding potential used for recording was 0 mV in all cases. Current amplitudes in response to the application of drugs were quantified using Clampfit 10.2 software. Passive holding currents were subtracted and the traces were filtered by a 100 Hz digital Gaussian low-pass filter.

### Experimental design and statistical analyses

For animal studies included in this work, no formal randomization protocol was applied but animals were arbitrarily allocated into each drug treatment group. A sample size minimum of four animals was selected based on previously performed power analyses on similar studies [38]. Animals were excluded if the data set collected was incomplete or the data were statistically significant outliers from the mean. Investigators were not blinded to treatment group assignments. Data were analyzed using *t*-tests and one and two-way repeated measure ANOVAs. Correlation results, *R*^2^, were assessed with regression analysis assessed using Graph-Pad Prism software. Data were normally distributed; variance was estimated and was found homogeneous among statistically compared groups (https://www.statskingdom.com/).

## Results

### Phasic changes in DA dynamics

Phasic, sub-second changes in DA dynamics, release and clearance rate, were examined using FSCV [20, 27, 39]. Figure 1 depicts examples of colorplots (Fig. 1A-C) of evoked DA release in response to medial forebrain bundle (MFB) electrical stimulation (60 Hz, 24 pulses, 4 ms, 180 µA) after i.p. administration of either vehicle (saline), cocaine (10 mg/kg or 29 μmol/kg), or JHW 007 (10 mg/kg or 24 μmol/kg), an atypical DUI [40, 41]. For each colorplot (Fig. 1A–C), the inset shows the cyclic voltammogram of DA recorded at the peak change in current, confirming DA detection. Above each color plot is the DA concentration profile shown as a function of time. Compared to saline (Fig. 1A), cocaine (Fig. 1B) produced a large increase in evoked DA_Max_ and a reduction in the rate of DA clearance. At variance with cocaine, JHW 007 (Fig. 1C) (24 μmol/kg) did not enhance evoked DA_Max_, but still decreased the clearance rate for DA.

### Reduction in DA clearance rate correlates with DAT affinity of DUIs

Figure 1D shows the effect of administration of DAT inhibitors on NAS DA clearance rate, as an index of DAT inhibition. Saline administration did not significantly modify DA clearance rate [paired *t*-test, two tail, *p* > 0.05 for each group] and no significant difference between the baselines of experimental groups was found [One-Way ANOVA *F* (5,21) = 1.6059, *p* = 0.20]. Dose-dependent, significant reduction in the clearance rate of DA was obtained after administration of typical DUI, cocaine, 1.5-165 μmol/kg [One-Way Repeated Measures ANOVA *F* (8,24) = 9.3706, *p* < 0.001], methylphenidate, 0.4–236 μmol/kg [One-Way Repeated Measures ANOVA *F* (10,30) = 31.7504, *p* < 0.001] and WIN 35428 (0.07–7.5 μmol/kg), and a dose-dependent significant decrease in the clearance rate of DA was also obtained after administration of the atypical DUIs JHW 007, 1.2–40 μmol/kg [One-Way Repeated Measures ANOVA *F* (6,30) = 4.3447, *p* < 0.01]; JJC8-091, 8–92 μmol/kg [One-Way Repeated Measures ANOVA *F* (5,20) = 9.5437, *p* < 0.001], and desipramine (10.6–330 μmol/kg) [One-Way Repeated Measure ANOVA *F* (7,21) = 5.2866, *p* = 0.001].

To determine if DAT affinity played a role in the function of each DUI, inhibition constants (IC_75_) for DA clearance rates (see the “Methods” section) were calculated from data in Fig. 1D and from data for *R*-Modafinil and two of its analogs, JJC8-016 and JJC8-088, previously studied under identical conditions [20]. Figure 1E shows a positive correlation (*R*^2^ = 0.826) between the IC_75_ and the log DAT affinity suggesting that the affinity of each inhibitor for DAT has a direct impact on its ability to reduce the clearance rate of DA from the extracellular space. Figure 1F shows the experimental setup with a recording electrode implanted in the NAS and a stimulation electrode positioned in the medial forebrain bundle.

### Typical and atypical DUIs differ in their ability to increase phasic, evoked DA_Max_

**Fig. 2:**
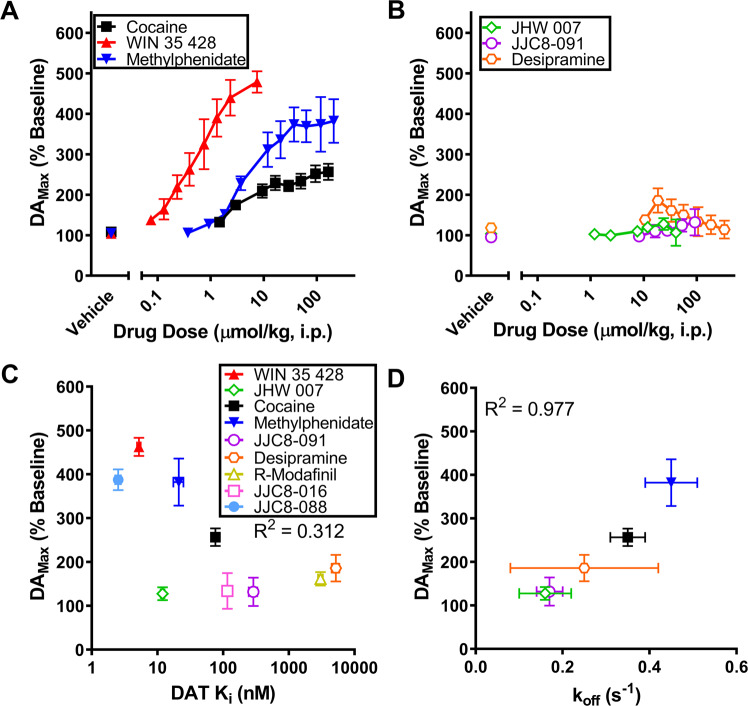
Typical and atypical dopamine uptake inhibitors differ in their ability to increase DA_Max_. **A** The stimulus-elicited DA_Max_ obtained after administration of typical DUI, cocaine (*n* = 4), methylphenidate (*n* = 8), and WIN 35428 (*n* = 4), is plotted as a function of drug dose. **B** The stimulus-elicited DA_Max_ obtained after administration of atypical DUIs, JHW 007 (*n* = 6), JJC8-091 (*n* = 5), and desipramine (*n* = 5), is plotted as a function of drug dose. From the data shown in (A) and (B), the maximal effect on evoked DA release was measured and plotted as a function of DAT affinity **C** for the series of DUIs included in panels (**A**) and (**B**) and for the DUIs, *R*-modafinil, JJC8-016 and JJC8-088, tested under the same experimental conditions in a recent publication [20], or as a function of DAT *k*_off_ rate (**D**) as measured by electrophysiology in HEK293-hDAT cells in the present manuscript or in recent literature [21] under the same experimental conditions. Error bars represent mean ± SEM.

Figure 2 shows cumulative dose–response effects for typical (Fig. 2A) and atypical (Fig. 2B) DUIs on evoked DA_Max_. Saline administration was found to have no significant effect on DA_Max_ [*t*-test *p* > 0.05 for each group], a One-Way ANOVA also shows that the baseline DA values did not significantly vary between groups [One-Way Repeated Measures ANOVA *F*(5,21) = 0.8932, *p* = 0.5]. The typical DUIs WIN 35-428 (0.07–7.5 μmol/kg) [One-Way Repeated Measures ANOVA *F*(8,24) = 38.41, *p* < 0.001]; methylphenidate (0.4–236 μmol/kg) [*F*(10,30) = 19.47, *p* < 0.001]; and cocaine (0.5–56 mg/kg) [One-Way Repeated Measures ANOVA F(8,24) = 11.906, *p* < 0.001] were each found to dose dependently increase evoked DA_Max_ to over 250% of baseline values (Fig. 2A). At variance with typical DAT blockers, only trends for dose-dependency on the enhancement of evoked DA_Max_ (lower than 30% and 90% over baseline) were obtained after administration of the atypical DUIs JHW 007 (1.2–40 μmol/kg) [One-Way Repeated Measures ANOVA *F*(6,30) = 1.9578, *p* = 0.1], and desipramine (10.6–330 μmol/kg) [*F*(7,21) = 1.463, *p* = 0.2], while JJC8-091 (8–92 μmol/kg) was found to produce a dose dependent effect [One-Way Repeated Measures ANOVA *F*(5,20) = 3.2988, *p* = 0.02], though its highest increase was not >30% over baseline values.

Figure 2C shows that no significant relationship (*R*^2^ = 0.312) was found between the highest change in DA_Max_ produced by each DUI and their DAT affinity, indicating that the ability of typical and atypical DUI to enhance evoked DA_Max_ was not related to the blockade of DAT. To provide a broader picture of the potential correlation, previously published data obtained with the administration of other typical and atypical DAT blockers, *R*-Modafinil, JJC8-016, and JJC8-088, under the same experimental conditions [20] were also included.

### The dissociation rate constant (*k*_off_) from DAT correlates with DUIs enhancement of stimulus-evoked DA_Max_

Figure 2D shows the relationship between DUIs enhancement of stimulus-evoked DA_Max_ measured by FSCV (from Fig. 2C) and their dissociation rate constant (*k*_off_) from DAT (the dwell time of each DUI in the DAT-binding site) [21] obtained in HEK293-hDAT cells. The figure shows a strong correlation (*R*^2^ = 0.977) due to DUIs with longer dissociation times and longer half-lives having a lower ability to increase the elicited DA peak, while cocaine and methylphenidate which are known to be quickly cleared from DAT have a shorter dissociation time and much greater impact on DA_Max_.

### Pretreatment with typical and atypical DUI’s differentially alters the effects of cocaine on phasic DA dynamics

**Fig. 3:**
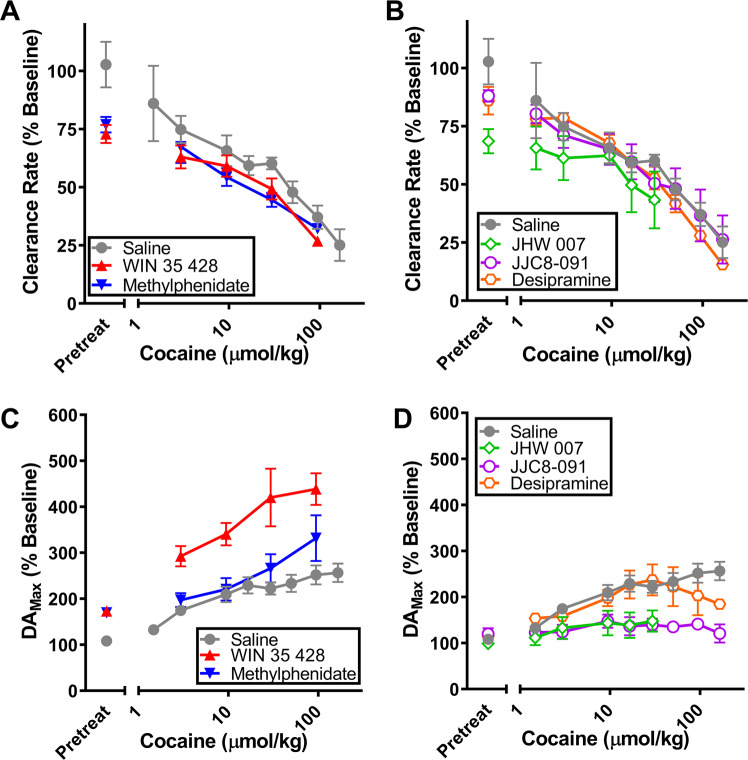
Pretreatment with typical and atypical DA uptake inhibitors alters the effects of cocaine on phasic DA. **A** Effects of pretreatments with the typical DA uptake inhibitors methylphenidate (*n* = 4) and WIN 35428 (*n* = 4) on changes in DA clearance rate induced by different doses of cocaine. **B** Effect of pretreatments with the atypical DA uptake inhibitors JHW 007 (*n* = 4), JJC8-091 (*n* = 6), and desipramine (*n* = 4) on changes in DA clearance rate produced by different doses of cocaine. **C** Effect of pretreatments with the typical DA uptake inhibitors methylphenidate (4.2 μmol/kg; *n* = 4) and WIN 35428 (0.23 μmol/kg; *n* = 4) on changes in stimulus-elicited DA_Max_ produced by administration of different cocaine doses. **D** Effects of the atypical DA uptake inhibitors JHW 007 (24 μmol/kg), JJC8-091 (16 μmol/kg), and desipramine (106 μmol/kg) on changes in stimulus-elicited DA_Max_ produced by different doses of cocaine. Baseline values were not found to significantly vary between groups for both DA_Max_ [One-Way ANOVA *F*(5,20) = 2.115, *p* = 0.105] and DA clearance rate [One-Way ANOVA *F*(5,20) = 1.4377, *p* = 0.25]. Error bars represent mean ± SEM.

Figure 3A and B show the effects of pretreatment with typical and atypical DUIs on DA clearance rate after administration of cumulative doses of cocaine. The left sides of panels A and B on Fig. 3 show that while saline did not significantly modify the DA clearance rate, pretreatments with WIN 35428, Methylphenidate, and JHW 007 significantly reduced the DA clearance rate before cocaine administration [*t*-test: *p* < 0.05]. In combination with cocaine, pretreatment with each DUI tended to further depress the clearance rate of DA as compared to saline pretreatment [Two-Way repeated measures ANOVA vs. saline pretreatment: **WIN 35428**, Drug F(1,6) = 16.098, *p* = 0.007, Dose *F*(4,24) = 28.52, *p* < 0.001, Interaction *F*(4,24) = 1.434, *p* = 0.25; **Methylphenidate**, Drug *F*(1,6) = 21.021, *p* < 0.001, Dose *F*(4,24) = 27.193, *p* < 0.001, Interaction *F*(4,24) = 1.083, *p* = 0.39; **JHW 007**, Drug *F*(1,6) = 5.312, *p* = 0.006, Dose *F*(5,30) = 5.6248, *p* = 0.005, Interaction *F*(5,30) = 0.954, *p* = 0.46; **JJC8-091**, Drug *F*(1,8) = 0.2261, *p* = 0.65, Dose *F*(8,64) = 30.6017, *p* < 0.001, Interaction *F*(8,64) = 0.2808, *p* = 0.97; **Desipramine**, Drug *F*(1,6) = 3.946, *p* = 0.05, Dose *F*(8,54) = 31.112, *p* = 0.001, Interaction *F*(8,54) = 0.582, *p* = 0.79].

Figure 3C and D show the effects of pretreatment with typical and atypical DUIs in combination with cocaine on evoked DA_Max_. Pretreatment with the typical DUIs WIN 35428 (0.23 μmol/kg) and Methylphenidate (4.2 μmol/kg) produced an increase in DA_Max_ near 170%, significantly higher than pretreatment with saline (see the left side of panel C, Fig. 3) [*t*-tests: *p* < 0.05]. In combination with cocaine, both drugs produced additive effects on DA_Max_. [Two-Way Repeated Measure ANOVA vs. saline pretreatment: **WIN 35428** Drug *F*(1,6) = 32.4115, *p* = 0.001, Dose *F*(4,24) = 27.7521, *p* < 0.001, Interaction *F*(4,24) = 1.434, *p* = 0.03; **Methylphenidate** Drug *F*(1,6) = 4.1417, *p* = 0.09, Dose *F*(4,24) = 21.4079, *p* < 0.001, Interaction *F*(4,24) = 1.8904, *p* = 0.14]. At variance with typical DUIs, combinations of JHW 007 (24 μmol/kg) or JJC8-091(16 μmol/kg) with cocaine tended to significantly blunt the effects of cocaine on evoked DA_Max_. [Two-Way Repeated Measure ANOVA: **JHW 007**, Drug *F*(1,6) = 5.2474, *p* = 0.006, Dose *F*(5,30) = 22.462, *p* < 0.001, Interaction *F*(5,30) = 4.7776, *p* = 0.002; **JJC8-091**, Drug *F*(1,8) = 38.4871, *p* < 0.001, Dose *F*(8,64) = 10.22, *p* < 0.001, Interaction *F*(8,64) = 0.2808, *p* < 0.001]. Desipramine (106 μmol/kg), the weakest DAT inhibitor tested, did not have a significant effect on the increase in DA_Max_ produced by cocaine. [Two-Way Repeated Measures ANOVA: Drug *F*(1,6) = 0.4662, *p* = 0.52, Dose *F*(8,48) = 12.25559, *p* < 0.001, Interaction *F*(8,48) = 1.4137, *p* = 0.22].

### Typical and atypical DUIs have different effects on free presynaptic calcium fluctuations

**Fig. 4:**
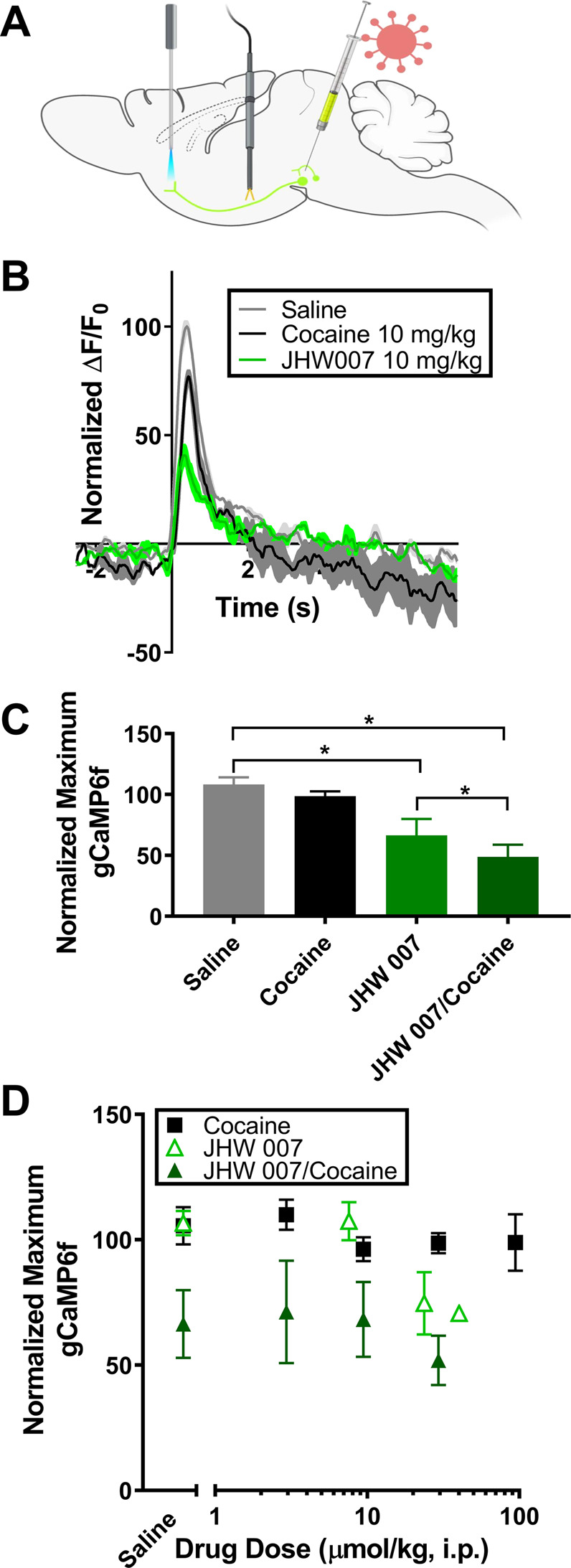
Typical and atypical DA uptake inhibitors have different effects on free presynaptic calcium. **A** Virus was injected into the VTA prior to the experiment. During the procedure an optical fiber was implanted in the NAc and a stimulating electrode in the MFB. See the “Methods” section for further detail. **B** Change in presynaptic free Ca^2+^ upon stimulus. Normalized, averaged time course of the change in presynaptic free calcium as measured by gCaMP6f upon electrical stimulation. Drugs were administered i.p. at least 20 min prior to measurement. **C** Effect of saline (*n* = 5) or drug treatment (29 μmol/kg cocaine, *n* = 5, or 24 μmol/kg JHW 007, *n* = 4, i.p.) on the electrically induced gCaMP6f peak. **D** The effect of saline, JHW 007 and cocaine on free presynaptic Ca^2+^ as a function of dose. Error bars represent mean ± SEM.

Figure 4A shows the experimental setup for the Calcium fluctuation tests in mice, where, prior to the experiment, the adeno-associated virus (AAV5) expressing GCaMP6f was injected into the VTA, an optical fiber was implanted in the NAS and a stimulating electrode was implanted in the MFB. As seen in Fig. 4B, in saline-treated controls free calcium (gray trace) rapidly increases in the NAS upon stimulus, then returns to baseline levels. A treatment of 29 μmol/kg cocaine (Fig. 4B, black trace) produces a similar effect on NAS-free calcium upon stimulus that was not found to be significantly different from saline treatment (Fig. 4C) [*t*-test *p* = 0.23]. An equipotent dose of JHW 007, 24 μmol/kg on evoked DA release was found to produce a free calcium peak in the NAS (Fig. 4B, green trace) significantly lower than that from saline treatment [*t*-test *p* < 0.05] (Fig. 4C).

In cumulative dosing studies identical to those for FSCV (Fig. 4D) cocaine (3–94 μmol/kg) showed no significant impact on free presynaptic calcium compared to baseline values [One-Way Repeated Measures ANOVA *F*(4,16) = 0.797, *p* = 0.54]. However, a cumulative dose response curve for JHW 007 (7.6–40 μmol/kg) showed a dose-dependent decrease in the maximum gCaMP6f peak (about 30%) after stimulation that was significantly lower than saline controls, [One-Way Repeated Measure ANOVA *F*(3.12) = 8.0825, *p* < 0.005].

Figure 4D also shows the effects of JHW 007 (24 μmol/kg) pretreatment on free presynaptic calcium fluctuations in the NAS in the presence of cocaine (3-94 μmol/kg). None of the cocaine doses tested was able to restore the decrease in free calcium peak produced by JHW 007 pretreatment to saline-like levels [Two-Way Repeated Measure ANOVA Drug *F*(1,7) = 16.93, *p* < 0.005, Dose *F*(3,21) = 2.3337, *p* = 0.1; Interaction *F*(3,21) = 0.8184, *p* = 0.5], suggesting a DAT inhibition independent mechanism of action in their effects on stimulated calcium peak.

### Inhibition of CaMKII by KN93 affects cocaine-induced enhancement in stimulus-elicited DA

**Fig. 5:**
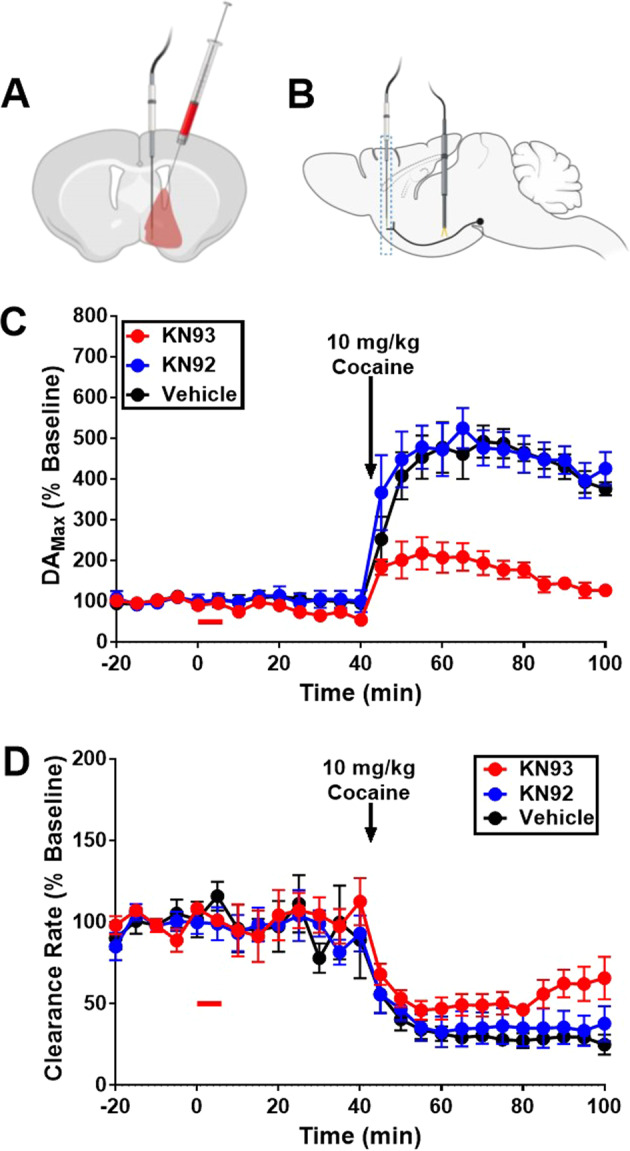
Inhibition of CaMKII by KN93 affects cocaine-enhanced evoked DA release. **A** Injections of KN92 (*n* = 4), KN93 (*n* = 4), or vehicle (*n* = 4) were made into the lateral ventricle, and the injected volume allowed to diffuse to nearby tissues shaded in red. **B** Placement of the working and stimulation electrode was identical to other experiments conducted in the present manuscript. **C** Effect of ICV administration of the vehicle (DMSO), KN93 (a CaMKII inhibitor) or KN92 (the inactive form of KN93) on stimulus elicited DA_Max_ as function of time, before and after administration of cocaine. **D** Effect of ICV administration of the vehicle, KN93 or KN92 on the clearance rate of DA as a function of time, before and after administration of cocaine. Error bars represent mean ± SEM, *n* ≥ 4 mice per group.

To investigate the potential involvement of CaMKIIα on cocaine-induced enhancement of evoked DA release, DA_Max_, local, intracerebroventricular injections of a CaMKII inhibitor, KN93 [28], an inactive analog, KN92, or vehicle (DMSO), were made into the ipsilateral lateral ventricle (Fig. 5A) during FSCV experiments (Fig. 5B). About 30 min following ICV injection, subjects were treated with cocaine, 29 μmol/kg (i.p.) (Fig. 5) and recording continued for an hour.

Phasic DA baseline values were not found to be significantly different between groups [One-Way ANOVA *F*(2,9) = 1.620, *p* = 0.25]. The effects of ICV treatment with KN93/KN92/vehicle administered before cocaine were found not to be significantly different from baseline [*t*-tests: *p* > 0.05]. The effects of KN92 as a pretreatment and after cocaine administration were not found to be different from vehicle [Two-Way ANOVA F(24,264) = 95.03, *p* = 0.97]. Pretreatment with the active CaMKII inhibitor, KN93, significantly blunted the effects of cocaine on DA_Max_ [Two-Way ANOVA F(24,264) = 12.4, *p* < 0.05] (Fig. 5C).

As seen in Fig. 5D, ICV injections of KN93, KN92, and DMSO did not significantly alter the clearance rate of DA, before cocaine treatment, compared to baseline values [One-Way ANOVA *F*(2,9) = 0.830, *p* = 0.47]. Additionally, pretreatments with KN93 or KN92 did not elicit effects significantly different from vehicle pretreatment on cocaine-induced decrease in DA clearance rate [Two-Way ANOVA *F*(24,264) = 0.99, *p* = 0.98].

## Discussion

The present results show that while all typical and atypical DUIs tested reduce DA clearance, suggesting their efficacy to inhibit DAT, only typical DUIs significantly enhance evoked DA release and produce additive effects when administered in combination with cocaine. On the other hand, atypical DUIs, when administered alone, show a limited, if any, enhancement of evoked DA release and, at variance with typical DUIs, they blunt the effects of cocaine on evoked DA release when administered in combination. We also found that cocaine enhancement of evoked DA release was significantly related to the dissociation rate constant, *k*_off_, from DAT, suggesting that DAT inhibitors with a faster dissociation (those that prefer to bind an outward facing DAT conformation) significantly enhance DA_Max_ at variance with DAT inhibitors with slower *k*_off_ (those that prefer to bind an inward facing DAT conformation) that showed a reduced, if any, effect on DA_Max_. Moreover, cocaine enhancement of DA_Max_ was blunted by ICV pretreatments with KN93, an inhibitor of CaMKII, suggesting a potential important interaction between this kinase and DAT involved in the dopaminergic actions of cocaine. Thus, our results suggest that different conformations of DAT stabilized by typical or atypical DAT blockers would favor, or not, its interaction with CaMKII. A CaMKII downstream activation of synapsins and mobilization of synapsin-dependent reserve pool of DA vesicles [31] would result in the enhancement of stimulus-evoked DA release by cocaine like typical DAT blockers, an effect blocked by ICV pretreatments with the CaMKII inhibitor KN93. Altogether, our results show that DAT conformational changes produced by its binding with typical or atypical DAT blockers differentially effects the evoked DA_Max_ and DAT’s interaction with CaMKII.

The efficacy of each drug as a DAT blocker is demonstrated by the significant and dose-dependent inhibition of DA clearance rate shared by all DUIs tested, which was significantly correlated with drug affinities for DAT, independently from their typicality. Cocaine and typical cocaine-like (WIN 35428, Methylphenidate) DUIs dose-dependently enhanced the stimulus-elicited DA release peak, as measured by DA_Max_. Cocaine in particular has been shown to increase the DA content of cytoplasmic DA containing LDCVs in rats [42], as well as rearranging the distribution of those vesicles in the neuron, affecting the exocytotic release of both DA and serotonin [26]. Cocaine effects on DA dynamics seem at variance with the atypical DUIs, JHW 007, JJC 8-091, and desipramine. These atypical DUIs, although binding to and blocking DAT, did not significantly affect the magnitude of the stimulus-elicited release of DA, which resulted in a non-significant correlation between DAT affinity and enhancement of evoked DA_Max_. Interestingly, this latter result is in agreement with the lack of consistent reinforcing effects previously reported for atypical DAT blockers in rodent models as compared to typical DUIs [16, 17, 40, 43, 44].

Our results show a relatively fast *k*_off_ from DAT [21] for typical DUIs. WIN 35428 was found to produce a dissociation rate too fast to be measured by this technique. Alternatively, the atypical DUIs were found to have much lower *k*_off_ values, suggesting longer interactions between these compounds and DAT before they could be dissociated. We also found a strong correlation between *k*_off_ and evoked DA release, with DUIs with longer dissociation times having a lower ability to increase DA_Max_, while cocaine and methylphenidate, which showed a shorter dissociation time, had a much greater impact on the maximal elicited DA peak. To explain these effects, we posit that typical DUIs largely associate with the outward-open conformation of DAT, in which the compound remains in close proximity to the surrounding extracellular fluid while the extracellular gate of DAT remains open [12, 45]. This is at variance with atypical DUIs that interact with a more inward-occluded DAT conformation, according to in silico modeling [15, 16] and molecular pharmacology experiments performed in mutated hDAT [18]. In this conformation the compound is less accessible to extracellular fluid and is bound to DAT deeper in the binding pocket, between the extracellular and intracellular gates. Also, in agreement with microdialysis data, the effects of some atypical DUIs, such as JHW 007, have been shown to produce longer lasting effects on extracellular DA levels than typical DUIs [17]. Moreover, a recent report shows that the cocaine-like DUI, nomifensine, promotes the rearrangement of DAT nanodomains distribution from nano-clusters characterized by an inward facing DAT conformation to a de-clustered distribution characterized by an outward-facing conformation of DAT [19]. Such rearrangement, which is at variance with that promoted by an atypical DUI, JHW 007, is also regulated by neuronal activity and calcium [19], suggesting a potential mechanism that promotes distribution and localization of DAT in the outward facing conformation on the neuronal membrane ready to provide an efficient DA reuptake in DA release active areas.

Pretreatments with typical or atypical DUIs differentially affected the consequences of cumulative doses of cocaine on DA dynamics. Our results show that all typical DUIs tested in combination with cocaine did not affect its ability to block DA reuptake, as shown by additive effects on DA clearance rate. Typical DUIs like WIN 35428 and methylphenidate produced additive effects also on cocaine-induced enhancement of evoked DA_Max_. In contrast, an opposite effect was observed for JJC8-091 and JHW 007, which blunted cocaine-induced enhancement of evoked DA_Max_. The mechanism behind this effect may relate to the conformation DAT assumes when interacting with an atypical DUI. The inward-occluded DAT conformation in addition to the slow DAT dissociation rate obtained with atypical DUIs may prevent DAT from binding to cocaine, thus preventing DAT conformational changes that lead to the enhancement effects of cocaine on evoked DA release.

Our results from fiber photometry tests provided a look at presynaptic calcium dynamics during the stimulus-evoked release of DA to determine if either type of DUIs had an effect on calcium dependency of exocytosis. While cocaine exposure did not significantly modify the stimulus-elicited intracellular calcium peak, the atypical DUI, JHW 007, dose dependently decreased the amplitude of the intracellular calcium peak. Subsequent cumulative dosing of cocaine did not significantly modify the decrease in free intracellular calcium peak elicited by JHW 007 pretreatment. In this report we have not explored the mechanism underlying the differences in changes of amplitude of the intracellular calcium peak elicited by typical or atypical DAT blockers. However, JHW007 has been shown to promote and stabilize an inward-facing DAT conformation in clustered nanodomains [19] that may impair calcium efflux, at variance with typical DUIs, suggesting another mechanism by which an atypical DUI may blunt the effects of cocaine. Indeed, the attenuating effects of JHW007 on intracellular calcium seem to correlate with other calcium dependent activities. For example, its lack of enhancement of evoked DA release, and its ability to prevent cocaine-induced increases in stimulus-evoked DA release.

Changes in intracellular free calcium levels can have a great effect on exocytosis, which would affect, for example, the storage of DA in LDCVs [46] that may be dependent upon calcium-sensitive molecular machinery. We have shown that KN93, an inhibitor of CaMKII, a calcium-dependent kinase known to interact with synapsin, synaptotagmin, DAT, and the D2 receptor [30, 47–49], blunts the effects of cocaine on stimulus-evoked DA_Max_. Importantly, ICV pretreatment administration of KN93 did not affect cocaine blockade of DAT, as shown by the similar magnitude of effects on the rate of DA clearance for cocaine in the presence of KN93, KN92 or vehicle, indicating that cocaine enhancement of evoked DA_Max_ may occur through a mechanism independent from DAT blockade.

In this work we have focused exclusively on DAT, a high-affinity DA transporter, but emerging work has revealed that there may also be a role for other membrane transporters in biological responses to addictive stimulants including the low affinity, high capacity OCT3 transporter [50–52]. Recent work has suggested that cocaine effects are not OCT3-mediated [53], in contrast to other psychostimulants [50], however, we acknowledge that exploring the role of other relevant transporters will be an important future direction of this work.

In conclusion, while all DUIs tested significantly reduced NAS DA clearance rate, an effect strongly correlated with DAT affinity, only typical DUIs significantly enhanced evoked DA release. No relationship was found between enhancement of DA_Max_ and DAT affinity, suggesting the involvement of alternative mechanisms in this effect in addition to DAT inhibition. Further, atypical DUIs blunted, while typical DUIs potentiated, cocaine-induced enhancement of DA_Max_ in the NAS. Cocaine has been suggested to enhance DA release by increasing mobilization of a synapsin-dependent reserve pool of DA vesicles [27]. Synapsin activity can be regulated by CaMKII [31], which also functionally interacts with DAT [29, 54]. We show that intracerebroventricular administration of an inhibitor of CaMKII activity, KN93, significantly attenuated cocaine effects on DA_Max_, but not its effect on DA clearance rate. Taken together, our results suggest that distinct conformations of DAT, stabilized by binding with typical or atypical DUIs, would facilitate or hinder DAT interactions with CaMKII, thus regulating phosphorylation of synapsins, mobilization of the reserve pool of DA vesicles and enhancement of DA release. Our results indicate that DAT might differently activate downstream effects (for example interaction with CaMKII) that are dependent on its different quaternary conformation stabilized/preferred by atypical versus typical DUIs that also may regulate DAT nanocluster distribution on DA terminals [19]. We suggest these downstream effects play a role in the dopaminergic effects of DUIs related to their psychostimulant actions and addictive liability. Finally, our data provide further evidence that atypical DUIs stabilize DAT conformations that differ from cocaine and play a significant role in blocking the neurochemical and behavioral actions of cocaine. The unique pharmacological profile of these atypical DUIs supports their therapeutic potential as medications to treat psychostimulant use disorder [10, 16].

## Data Availability

All data included in the present manuscript are available within the article or will be made available upon request.

## Code availability

Matlab code to extract and preprocess photometry data will be made available upon request.
